# Features and effects of computer-based games on cognitive impairments in children with autism spectrum disorder: an evidence-based systematic literature review

**DOI:** 10.1186/s12888-022-04501-1

**Published:** 2023-01-03

**Authors:** Sorayya Rezayi, Mehdi Tehrani-Doost, Leila Shahmoradi

**Affiliations:** 1 Ph.D. Candidate of Medical Informatics, Health Information Management and Medical Informatics Department, School of Allied Medical Sciences, Tehran University of Medical Sciences, Tehran, Iran; 2 Professor of Child and Adolescent Psychiatry, Research Center for Cognitive and Behavioral Sciences, Tehran University of Medical Sciences, Tehran, Iran; 3 Department of Psychiatry, Roozbeh Hospital, Tehran University of Medical Sciences, Tehran, Iran; 4 Full Professor, Health Information Management and Medical Informatics Department, School of Allied Medical Sciences, Tehran University of Medical Sciences, Tehran, Iran

**Keywords:** Computer Game, Autistic Disorders, Cognition Therapy, Children

## Abstract

**Introduction:**

Children with Autism Spectrum Disorder (ASD) have different cognitive and intelligence profiles than typical developing individuals. Some of these children need cognitive rehabilitation. This study's main purpose is to provide a systematic review about applying computerized cognitive games for autistic children and to determine the effectiveness of such interventions.

**Material and methods:**

A thorough search of the ISI Web of Science, Medline (through PubMed), Scopus, IEEE Xplore, and APA PsycInfo databases was performed for articles published from inception to May 17, 2022.

**Results:**

Of 1746 papers, 28 studies were found to be eligible in this systematic review. Fifteen studies (53.57%) compared a Control Group (CG) with Experimental Groups (EGs), while 13 papers (46.42%) evaluated only the impact of the applied intervention in an experimental group. Major domains of cognitive functions are divided into five main categories: 1. Executive functions, 2. Social cognition/emotions, 3. Attention/concentration, 4. Learning and memory, and 5. Language. In 42.85% (12 studies) of the screened papers, social cognition and emotions were assessed after cognitive rehabilitation. The highest rate of effects reported by studies were related to social cognition enhancement. Of the total number of included studies, 17 studies reported a positive effect at all scales, of which nine were quasi-experimental, and seven were fully experimental.

**Conclusion:**

Using suitable computerized game-based solutions could enhance cognition indexes in autistic children. Hence, further investigation is needed to determine the real effectiveness of these novel technologies.

**Supplementary Information:**

The online version contains supplementary material available at 10.1186/s12888-022-04501-1.

## Introduction

Autism Spectrum Disorder (ASD) is a lifelong neurodevelopmental condition distinguished by abnormalities in communication, language, typical cognitive components, fun activities/play, reciprocal social interaction, restricted and repetitive behaviors [1]. According to the Diagnostic and Statistical Manual of Mental Disorders (DSM-5), ASD is a group of changes that emerge between 12 and 14 months of age [2]. Studies show that the prevalence of ASD has increased in recent years. The Centers for Disease Control and Prevention (CDC) reported in 2022 that approximately 1 in 44 children in the United States is diagnosed with autism [3]. Autism prevalence has increased 178% since 2000. According to 2021 statistics, 1 in 34 boys and 1 in 144 girls are identified with autism, and boys are four times more likely to be diagnosed with autism than girls [4].

Researchers have explored the cognitive profiles of autistic individuals for decades [5]. Children with ASD have different cognitive and intelligence profiles than typical developing individuals [6]. Research findings demonstrate a high rate of uneven cognitive development in children with ASD [7]; some support distinct cognitive profiles in ASD and may provide further evidence for distinct etiological mechanisms [8]. Furthermore, several studies have suggested that children with ASD have delayed cognitive skills [7, 9, 10]. Likewise, there is a study that has investigated the cognitive profiles of infants and toddlers on the spectrum, and there is an uneven and poor cognitive profile of abilities [11]. These children also have apparent deficits or delays in daily adaptive activities [6, 12]. The brains of autistic children need to return to or reach the optimal point [13, 14]. Therefore, individuals with ASD have atypical cognitive functions, including executive dysfunction, atypical perceptual, impaired social cognition and perception, and information processing, which is usually correlated with attentional deficiencies [15, 16]; it can be acknowledged that such deficits can be enhanced by cognitive rehabilitation techniques [17, 18].

According to studies and DSM‑5, six major cognitive function domains are as follows: 1. Executive functions, 2. Social cognition/emotions, 3. Language, 4. Attention/concentration, 5. Visuospatial and motor function, and 6. Learning and memory [19–21]. Each of these six main areas has sub-sections that are also mentioned in various studies. Not all researchers agree on the classification of cognitive functions, however, and slight differences in the classification exist; nonetheless, the nature of all classifications is the same. Cognitive rehabilitation includes a set of targeted programs that are used to restore or improve one, several, or all of the six beforementioned cognitive functions [22]. More comprehensively, cognitive rehabilitation therapy focuses on restoring, strengthening, and intensifying cognitive functions (six functions and their sub-parts) that are impaired because of brain damage, stroke, or congenital disorder [23]. Scientifically speaking, cognitive rehabilitation treatment can lead to the improvement of cognitive abilities in children with ASD with different degrees of disability [12].

Applying Information and Communication Technologies (ICTs) can compensate and encourage the treatment of children with special requirements and ASD [24]. ICTs and novel computerized approaches make it possible to create controllable, predictable environments like games; they offer multisensory stimulation, which is ordinarily visual [25]. These technologies promote the ability for self-control and to work independently [26]. Consequently, among the proliferation of computers and the Internet, applying computer games, both offline and online, has symbolically increased [27]. With the increase in the capabilities of personal computers and mobile phones, the invention of personal mobile systems (tablets) and the provision of computer games on these systems, the use of computer games has broken the boundaries of time and place [28]. Cognitive games are specifically designed to reinforce cognitive characteristics; however, games that are specially utilized to evaluate or reinforce cognitive components focus on one or more cognitive components and reinforce them [29].

By using modern rehabilitation and cognitive empowerment methods, some of the cognitive abilities of ASD children are improved, such that they can move in the best direction in adulthood and suffer the least socio-psychological impairment [30]. Among these emerging interventions, the use of game-based tools for cognitive rehabilitation is rapidly increasing [22]. Computer-based games may be effective in improving cognitive and social skills in children with ASD, and there is good evidence of their efficacy in individuals with ASD. These games target cognitive problems such as attention, short-term and long-term memory, eye-hand adaptation, executive functions, daily functions, processing, and learning. Computerized games can be more engaging than routine exercises, because they replace reward and motivation systems with real-world motivations as a complement to rehabilitation activities. People can be immersed in the game world, and their ability and knowledge can be improved without any danger [31, 32].

In recent years, reviews concerning ASD and computerized-based approaches have made some contributions. Shahmoradi et al. provide a review of virtual reality interventions in cognitive rehabilitation of people with ASD [33]. A scoping review was conducted in 2022 and concentrated on studies utilizing virtual reality and augmented reality technology in social skills interventions for individuals with ASD. The main difference between previous work and ours is that previous studies have focused on interventions based on virtual and augmented reality and considered only the social cognition of autistic people [34]. In 2017, Liu et al. performed a review of the technology-facilitated diagnosis and treatment of ASD. This study differs from ours in many aspects, but the main difference is that their review focuses on the engineering perspective of autism studies [35]. In 2017, van Bennekom et al. conducted a review in which they evaluated the assessment of psychiatric disorders by means of a virtual reality environment; however, they did not focus on ASD [36]. A systematic review was published in 2022 by Shahmoradi. et al. In this contribution, the authors provide an overview of applying serious games in attention rehabilitation in patients with traumatic brain injury. This review, however, focused specially on other disorders, not ASD [31]. Patricia Mesa-Gresa et al. published a systematic review that focused mainly on the effectiveness of virtual reality for children and adolescents with ASD. Their study differs from ours mainly in that the previous researchers focused only on virtual reality environments and targeted a different age range [37].

The current systematic review aimed to document experimental investigations on the efficacy of computerized game-based cognitive interventions in children with autism and to evaluate their study design and methodology. We present considerations for experts and clinicians when utilizing games to remediate cognitive deficits. Our contribution has another important added value: In addition to investigating the effect of using computer games on the cognitive components of autistic children, all articles included in this systematic review were examined and compared from various aspects such as bibliometric data. Moreover, because we searched most databases to retrieve articles, our contribution covers a wider range of publications, offering a very thorough review.

### Objectives

This qualitative literature review outlines findings about computerized games in cognitive rehabilitation for children with ASD. The leading queries and ambiguities of this review are as follows:

1. Generally, how many articles investigating the impact of computer-based games on the cognitive functions of autistic children have been published (what is the publication trend)?
2. What bibliometric data do these studies include (journal level, country, publisher, and keyword frequencies)?
3. What are the major characteristics of the papers, i.e., the name and type of game, sample size, cognitive consequences of the goal, evaluation, and reported limitations/challenges?
4. How successful have computerized games been reported in improving the cognitive functions of ASD children?

## Materials and methods

### Information sources

The current review was performed according to the Preferred Reporting Items for Systematic Reviews and Meta‐analysis (PRISMA) statement [38]. Research presenting information on cognitive interventions while practicing games with autistic children population was distinguished. To do this, a comprehensive literature search of Medline (through PubMed), Web of Science (WOS), APA PsycInfo, Scopus, and IEEE Xplore was conducted for English articles published up to May 17, 2022 (without time limitation). A combination of Medical Subject Headings (MeSH) and Emtree keywords and terms related to autism, cognitive disorders, game, and children were applied in the search strategy (Appendix Table S1).

### Study selection

The selected academic papers were screened based on the exclusion and inclusion criteria provided bellow:

#### Inclusion criteria

Setting the correct research question in the determination criteria is notable in discovering more relevant evidence in the literature. Accordingly, the PICO model was selected for this purpose. A reliable and exhaustive question should comprise four parts that recognize the patient problem or population (P), intervention (I), comparison (C), and outcome(s) (O) [39]. The following PICO question was organized for the research determination process:

- Population— Preschool Children or Children (age < 13) with ASD.
- Intervention—Computer-based game interventions (to improve various complex cognitive functions, we have selected five main domains of cognitive functions: 1. Executive functions, 2. Social cognition/emotions, 3. Language, 4. Attention/concentration, and 5. Learning and memory).
- Comparison—(versus) Non-game-based interventions (children’s condition before game-based interventions, without rehabilitation: intergroup or baseline comparisons were conducted).
- Outcome—Critical outcomes reached.

#### Exclusion criteria

o Language other than English,
p Full abstract not available for review,
q Decentralized studies on autism disorder,
r No use of any computer games in study methodology,
s Book chapters, reviews, dissertations, meta-analyses, letters to editors, short briefs, short papers, and commentaries,
t Interventions without effectiveness reports,
u Articles unrelated to the main five cognitive functions were removed.

### Data extraction and screening

According to the high coverage of electronic databases such as PubMed, Web of Science, Scopus, and IEEE Xplore, all three authors decided to select these databases, and based on a comment by MT, APA PsycInfo was added. In searches of scientific databases (PubMed, Web of Science, Scopus, APA PsycInfo, and IEEE Xplore) with no time limitations for articles, a total of 1746 papers were retrieved. Manual searches to ensure the inclusion of all related papers and reduce the possibility of bias were also performed using two approaches: (1) checking the references of related papers, and (2) utilizing the Google Scholar search engine. After duplicates were removed, 1241 citations remained. Some exclusion and inclusion criteria were adjusted for screening-related citations in Sect. 2.2. Three authors (SR, LS, and MT) independently mined all abstracts and titles of the retrieved papers to include all eligible studies; LS supervised this round. Next, the full texts of relevant papers were screened, mined, and checked thoroughly by two authors (SR and LS) under the supervision of MT. Before data was extracted from the full text of the articles, an inter-author reliability check was performed. At this stage, two authors randomly selected 50% of the included articles and 20% of the excluded articles and checked the reliability between them. All of the extracted features and general information were re-checked for the authors to reach an agreement. All qualitative analyses were conducted in SPSS v20, and EndNote X9 and VOSviewer were applied for resource management.

### Analysis and synthesis phase

**Fig. 1:**
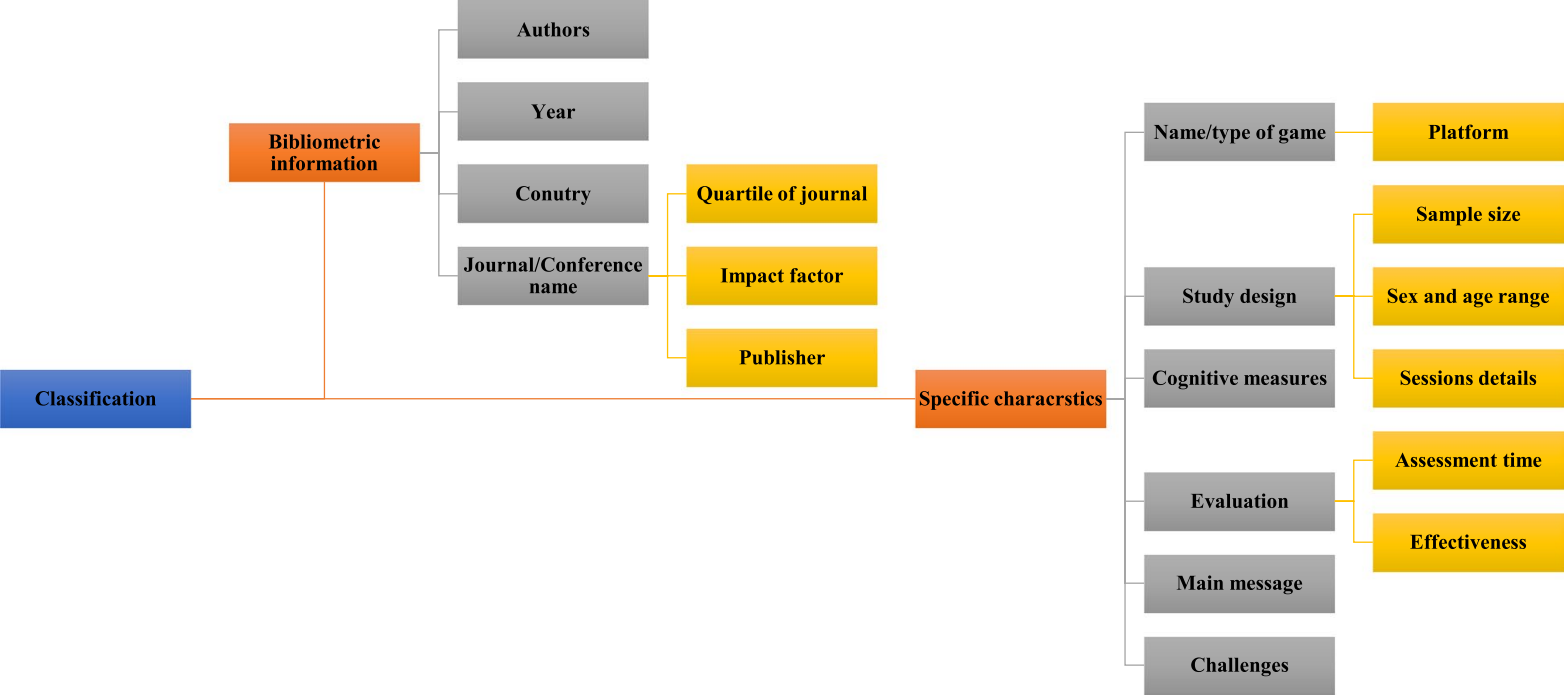
The classification of reviewed articles

Because of the heterogeneity of the papers in terms of results and statistical analyses, no meta-analysis was performed. The critical characteristics of each study were extracted for use in narrative syntheses. The authors summarized the main characteristics, and they are presented in Fig. 1. The effectiveness of computerized game-based interventions was classified as positive without statistical argument, statistically significantly positive, or no effect (not statistically significant). Study designs were categorized into two classes: true experimental design and quasi-experimental designs. Randomized Controlled Trial (RCTs) is an example of true experimental design, and quasi-experimental designs include two main designs: 1) Non-randomized Controlled Clinical Trial (NCCT), a nonrandomly assigned control (or comparison) group, and 2) time-series design with pre- and posttest comparisons (before and after clinical trial without a control group) [40]. Some metrics for the included studies were also analyzed, including year of publication, journal source, journal ranking, and the Impact Factor (IF) of all journals obtained from the Journal Citation Reports of 2020.

### Risk of bias and quality assessment

The Effective Public Health Practice Project (EPHPP) quality appraisal tool was applied to assess the methodological quality of all included citations [41]. The EPHPP is a reliable and appropriate tool for assessing different study designs, such as randomized controlled trials and non-randomized controlled trials. In this phase, three authors independently carried out the quality rating by the EPHPP scale; any disagreements were resolved by discussion. Each criterion was graded as strong, moderate, or weak, and then the overall quality score, i.e., global rating, was measured for each study. Studies with two or more weak ratings were given a global rating of “weak,” studies with one weak rating were given a global rating of “moderate,” and studies with no weak ratings were given a global rating of “strong” [33].

## Results

### Study selection

**Fig. 2:**
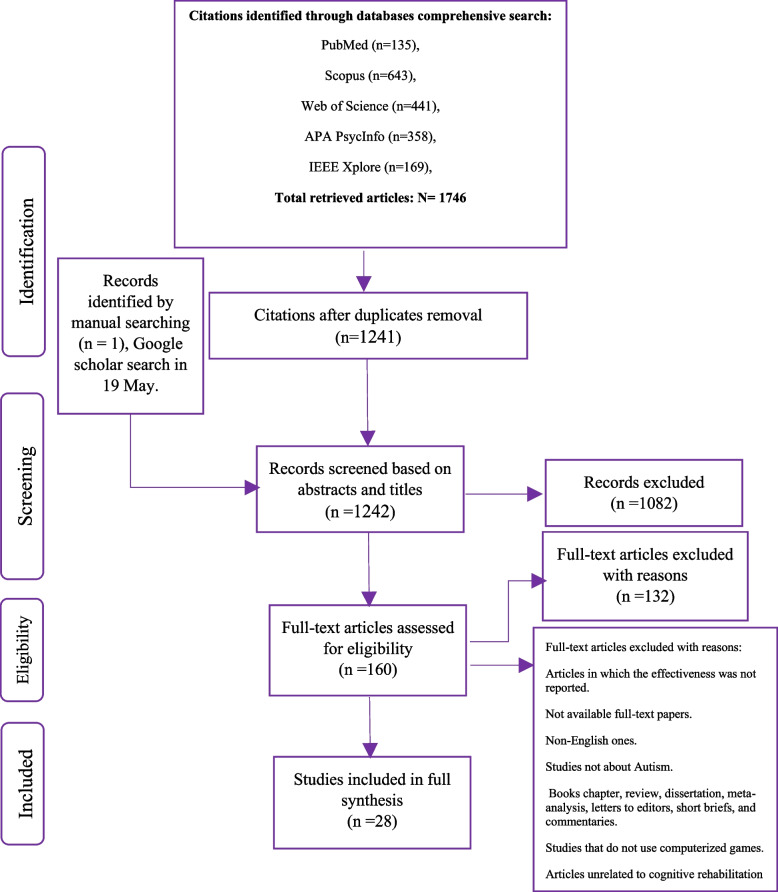
The PRISMA diagram for the records search and study selection

The process of searching scientific databases and identifying papers based on the PRISMA statement is presented in Fig. 2. A total of 1746 papers were retrieved. After duplicates were removed, 1241 articles remained for screening. Title and abstract screening led to the deletion of another 1082 articles. Fifty articles seemed relevant in the first phase, and their full texts were scrutinized. After reviewing the full texts of the articles and applying the inclusion and exclusion criteria, 28 studies were included in our systematic review. A summary of the key results is described in Table 1.

**Table 1:** Summary of included contributions (*n* = 28)

Contribution	Journal/Conference name	Name/Type of games used for intervention	Platform	Study design	Final group characteristics: Sample size: Groups (Sex)/Age	Procedure details (session number, duration and frequency)	Targeted cognitive measures	Assessment time	Evaluation scales	Evaluation	Effectiveness	Main message
Alvares GA et al., 2019, Australia [42]	Autism Research	Serious Game	Android playstore	Randomized controlled trial	N = 56 EG: 28 (22 M, 6 F) CG: 28 (26 M, 2F) Age: 5–12 yrs	1 session, 15 min	• Social interaction skills	Baseline and post intervention	• SRS-2, •MSEL • WISC-IV	Pairwise comparisons confirmed increased proportion of social characters selected in the training group across all levels (ap-values < 0.001); Children in the training group significantly raised the percentage of engagements to faces relative to objects after training. The adjusted mean change is 17.24 for the training group, and adjusted mean change is − 12.87 for the control group	Statistically significant at all evaluation scales	Findings indicated that training-based paradigms can improve statistically target fundamental attentional processing of social information in ASD
Chen J et al., 2019, China [43]	Computer Assisted Surgery	FaceSay: Serious Game	PC	Before & After Clinical Trial (without control)	*N* = 11 EG: 11 Age: 6–12 yrs	5 sessions, 29 min, once a week	• Emotional Skills	Baseline and at the end of the last week of intervention	• SRS-2	The results show that there is a non-significant difference between SRS total score at baseline and endpoint, with Chi-sq. 0.28, *p* value 0.60	Not effective	In short-term therapy, there is no improvement in social cognition, social awareness and social communication
Özen A, 2015, Turkey [44]	Educational Sciences: Theory & Practice	Video game	IOS apple	Non-randomized Controlled Clinical Trial	*N* = 6 EG: 3 (1 M, 2 F) CG: 3 (2 M, 1 F) Age: 5–11 yrs	15 sessions, 40 min, twice a week	• Social interaction skills	Baseline, the first and the second week after the intervention	• SRS-2	The analysis shows that one of the participants performed social interaction skills with 85% accuracy, another person with 91% accuracy, and the third person with 88% accuracy. The 4th, 5th, and 6th person performed the skill of responding appropriately to teaching opportunities with more than 80% accuracy in the intervention sessions	Statistically significant at all evaluation scales	The data suggest that participants can learn how to use the social interaction skills necessary for iPad gaming activities
Fantasia V et al., 2020, Italy [45]	Autism	Serious Game	Android playstore	Before & After Clinical Trial (without control)	*N* = 29 EG: 29 (25 M, 4 F) Age: 6–12 yrs	2 sessions, once a week	• Short-term memory • Long-term memory	Baseline and the first half of the study phase and after 1-week later intervention	• PPVT-R • VABS-II • RCPM	Results showed that memory-based recognition accuracy was high for objects studied in the active condition in both the test (active: M = 0.63, SD = 0.20; yoked: M = 0.53, SD = 0.20) and the retest (active: M = 0.54, SD = 0.22; yoked: M = 0.48, SD = 0.20)	Statistically significant at all evaluation scales	Advancements in active study status continued, especially for one week after the initial study session
Aresti-Bartolome N and Garcia-Zapirain B, 2015, Spain [46]	Bio-medical materials and engineering	Serious videogame	PC	Non-randomized Controlled Clinical Trial	*N* = 40 EG: 20 CG: 20 Age: 3–8 yrs	2 sessions, 12 min	• Attention • Social interaction skills	Baseline and end of each game session	• Researchers set scales (Errors, Response time, Interaction with visual contact, Interaction without visual contact, and Gaze and pupil information during the game)	The reaction time of the first group was lower after rehabilitation sessions (M = 4.52 s, SD = 3.40); the clinical group (M = 36, SD = 38.18); the response time decreased when the clinical group provided interaction with eye contact	Statistically significant on some evaluation scales	The results showed that the interaction involving looking at the eyes improved as the reaction time decreased, indicating that the system can help with cognitive rehabilitation
Almeida LM et al., 2019, Brazil [47]	International Journal of Computer Games Technology	ALTRIRAS	PC	Non-randomized Controlled Clinical Trial	*N* = 38 EG: 10 CG: 28 Age: 6–12 yrs	4 sessions, 60 min, once a week	• Facial processing skills • Social interaction skills	Baseline and post intervention	• Researchers set scales (QuizEmotion scale)	Based on the applied statistical test, there was no significant difference between the number of correct answers of children with ASD in the pre- and post-test	Not effective	Play exposure time in children with ASD should be increased to effectively aid facial expression recognition
Fernandes FD et al.,2010, Brazil [48]	PRO-FONO: Revista de Actualizacao Cientifica	Not reported	PC	Before & After Clinical Trial (without control)	*N* = 23 EG: 23 Age: 3–12 yrs	10 sessions	• Language skills	Baseline and after each session	• Functional communicative Profile /scales	Statistical analysis has not identified a significant difference (0.05) with any variables, but the number of subjects and areas with progress can be considered	Not effective	Quantitative and qualitative improvements were identified without statistical significance. This progress was observed after a shorter period than is usually applied to this type of comparison, which seems favorable
Al-Hammadi M andAbdelazim A, 2015, USA [49]	In2015 IEEE Global Engineering Education Conference	Miss.Fly: Video game	PC	Partially Randomized clinical trial	*N* = 98 EG: 49 CG: 49 Age: 6–7 yrs	Not reported	• Attention • Short-term memory • Long-term memory	End of each game session	• Researchers set scales: (Time response (seconds), Accuracy, Pay Attention, Strengthen Memorization, React to Random Processes)	The independent samples t-test was associated with a statistically significant effect since *p* < 0.05. Then, the autistic children’s performance does differ significantly; the independent samples t-test was associated with a statistically significant effect since *p* < 0.05, t (35) = 4.02, *p* = 0.002. Thus, the autistic children’s time response does differ significantly	Statistically significant at all evaluation scales	The designed game improves the ability of children of both groups to pay attention, remember, react to random processes, and process parallel information
Pedreschi VB et al., 2019, Peru [50]	Virtual Reality	Serious Game	Android playstore	Before & After Clinical Trial (without control)	*N* = 20 EG: 20 Age: 3–10 yrs	During 2-weeks	Emotional Skills Social interaction skills Facial processing skills	Baseline and post intervention	• Researchers set scales: (Emotion Recognition Times)	The findings demonstrate that 67% of the ASD patients enhanced their emotion recognition skills (represented in caricatured and human facial expressions)	Positive without statistical argument	The outcomes revealed a significant advancement in emotion recognition after employing the Serious Game
Bono V et al., 2016, UK [51]	Frontiers in psychiatry	GOLIAH: Serious Game	PC and Mobile app	Before & After Clinical Trial (without control)	*N* = 10 EG: (10 M, 0 F) Age: 5–9 yrs	60 sessions, during 3-month, 20 min	• Attention • Imitation skills	Baseline, after each session and post 3-month intervention	• WPPSI • VIQ • PIQ	The time to terminate the assignment significantly declined along sessions (*p* < 0.001); correct answers raised considerably with the session numbers (*p* = 0.005); the quality of imitation enhanced throughout the sessions	Statistically significant at all evaluation scales	The game platform of this study is useful both in the child-therapist interaction in the hospital and in the child-parent interaction at home
Kamaruzaman NN et al., 2017, Malaysia [52]	Indian Journal of Science and Technology	Quranic: Serious game	Android playstore	Before & After Clinical Trial (without control)	*N* = 15 EG: 15 (13 M, 2 F) Age: 5–12 yrs	Not reported	• Learning • Attention • Imitation skills	Baseline and post intervention	• Researchers set scales: (Involvement Scale, Reaction Time, Facial Expression & Posture, Complexity and Creativity)	There was a significant effect of SDs of signals in the involvement scale (*P*-value < 0.05); the level of student engagement in learning Al-Quran improved after using the Quranic game	Statistically significant at all evaluation scales	The upshots pointed out that the involvement of children in playing games influences the level of engagement, as the analysis suggests a significant relationship between involvement and engagement level
Jeekratok K et al., 2014, Thailand [53]	International Journal of Web-Based Learning and Teaching Technologies	Video game	PC	Before & After Clinical Trial (without control)	*N* = 10 EG: 10 Age: 7–10 yrs	36 sessions, during 3-months, 60 min	• Attention Imitation skills Recognizing and differentiating	Baseline and post intervention	• S-CAT • PECS • Researchers set scales	Average post-test scores were higher than pre-test scores (Z = 2.81, 2.81 and 2.80 respectively); the t-values of the three variables were significantly different at *p* < .01	Statistically significant at all evaluation scales	There is robust evidence to support the hypothesis that web-based games and social stories can be efficacious as instruments for behavior changeover
Bernardini S et al., 2014, UK [54]	Information Sciences	ECHOES: Serious game	PC	Before & After Clinical Trial (without control)	*N* = 19 EG:19 (18 M, 1 F) Age: 7- 13 yrs	several times a week over a 6-week, 10–20 min	• Social interaction skills • Facial processing skills • Language skills	Baseline inter and post intervention	• Researchers set scales	The mean probability of children responding to the practitioner’s bids for interaction during the pre-test was 0.66 and during the post-test was 0.71 (SD = 0.14); this slight increase in responses between the pre and post-test was not significant. Across the sessions, by some children, the results showed a slight but non-significant decrease	Statistically significant only for some children	Empirical marks of the agent's effectiveness are based on an extensive evaluation of the ECHOES platform, which shows encouraging tendencies for several children
Mairena MÁ et al., 2019, Spain [55]	Research in Autism Spectrum Disorders	Pico’s Adventure: Video game	PC	Randomized controlled trial	*N* = 15 EG: 7 (7 M, 0 F) CG: 8 (8 M, 0 F) Age: 4–7 yrs	4 sessions, 30 min	• Social interaction skills	Baseline and post intervention	• Researchers set scales: (Social Initiation, Spontaneous Gestures, Responses, Interlocutor, Given helps, Other social Interaction), • ASEBA • ABC • SSRS	Children demonstrated significantly more social initiation during the videogame (M = 9.33, SD = 9.61) than during free Play (M = 4.08, SD = 3.82); t (11) = 2.438, *p* = 0.033. Besides, outcomes associated with repetitive actions indicate the prospect of this game to lessen repetitive behaviors	Statistically significant on some evaluation scales	There is s potential use of full-body interactive videogames as tools to foster social initiation conducts in children with ASD
de Vries M et al., 2014, Netherlands [22]	Journal of Child Psychology and Psychiatry	Braingame Brian: Video game	PC	Randomized controlled trial	*N* = 90 EG1: 31 EG2: 27 CG: 32 Age: 8–12 yrs	25 sessions, one a week, 40–50 min	• Working memory • Facial processing skills	Baseline, post intervention, and 6-week-follow-up	• BRIEF • SART • Corsi-BTT	Children in the WM and flexibility intervention-conditions improved significantly in sequence lengths in the WM-training tasks, and level in the flexibility-training task (*p*’s < .001.(	Statistically significant at all evaluation scales	All children are enhanced in working memory, flexibility, attention, executive functions, social behavior, and quality of life. However, the adaptive intervention conditions did not result in a more considerable improvement than the mock training
Saniee S et al., 2019, Iran [56]	Journal of Intellectual Disability Research	Tatka: Video game	PC	Before & After Clinical Trial (without control)	*N* = 13 EG: 13 (11 M, 2 F) Age: 5–7 yrs	4 sessions, 15-min per day during 2-months	• Set-shifting ability	Baseline, post intervention, and 6-week-follow-up	• SSIT • WCST • MCST	According to the intervention findings; a significant difference in BFRS-R was observed between pre-training and post-training (*P* = 0.0001)	Statistically significant at all evaluation scales	All children improved considerably in cognitive and behavior flexibilities when they were given the SSIT
Khowaja K and Salim SS, 2019, Malaysia [57]	International journal of human–computer interaction	Serious Game	PC	Before & After Clinical Trial (without control)	*N* = 5 EG: 5 (5 M, 0 F) Age: 6–10 yrs	15 sessions, 20 min, during 1-months	• Learning	Baseline, post intervention, and week one and week two following the withdrawal of intervention (follow-up)	• Researchers set scales: (Correct responses, Number of attempts) • SSIT	The use of SGs during intervention improved the performance of participants 1, 2, 3, 4, and 5 to 97%, 94%, 81%, 96%, and 89%, respectively	Statistically significant at all evaluation scales	The results indicated that learning vocabulary entities among children with ASD enhanced after using the game
Fridenson-Hayo S et al., 2017, Israel [58]	European child & adolescent psychiatry	Emotiplay: Serious Game	PC	**Phase 1:** Before & After Clinical Trial (without control) **Phase 2:** Randomized controlled trial with two intervention groups and controls	N (1) = 15, EG 1: 15 (11 M, 4 F) N (2) = 74 EG (2): 34 CG (2): 40 Age: 6–9 yrs	At least 2 h per week, over a period of 8 weeks	• Emotional Skills • Facial processing skills • Vocal intonation • Body language	Baseline and post intervention	• SRS-2 • VABS-II • Emotion Recognition Tasks	**Phase 1:** SG use significantly improved participants’ performance on the ER body language task (Pre: M = 14.33, Post: M = 18.73, *p* < .01) and the ER integrative task (Pre: M = 11.13, Post: M = 13.47, *p* < .05) **Phase 2:** Pairwise comparisons for the time by group interaction demonstrated that considerable improvement over time was found on all ER tasks for the intervention groups (Face: Mean difference = 2.17, *p* < .001; Voice: Mean difference = 2.19, *p* < .001; Body: Mean difference = 4.63, *p* < .001)	Statistically significant at all evaluation scales	This game (Emotiplay’s SG) is an influential and motivating intervention, cross-culturally leading ER from faces, voices, body language, and their integration in context to children with high-functioning ASC
Spaniol MM et al., 2017, Brazil [32]	Journal of Autism and Developmental Disorders	CPAT	PC	Randomized controlled trial	*N* = 14 EC: 8 (6 M, 2 F) CG: 6 (6 M, 0 F) Age: 6–10 yrs	13 sessions, 45 min, twice a week across a 2-month period	• Attention • Academic performance	Baseline (1 and 2 weeks before) and post intervention	• CPM • Academic Assessments (Maths, Reading Comprehension, Copying) • CARS	Effects showed a significant increase in the CPM scores from pre to post-assessment for the CPAT group (pre = 86.25 ± 6.73; post = 100 ± 5.98); there was a significant improvement in math scores for the CPAT group (pre = 27.8 ± 10.14; post = 51.7 ± 9.98); there was a significant improvement in reading scores for the CPAT group (pre = 39.34 ± 6.19; post = 59.19)	Statistically significant at all evaluation scales	Progress was evident in various academic tests, including reading comprehension, copying speed, and math
Hu X et al., 2019, China [59]	Journal of Autism and Developmental Disorders	CAI	PC	Before & After Clinical Trial (without control)	*N* = 4 EG: 4 (3 M, 1 F) Age: 9–11 yrs	7–13 sessions, 20 min during 5 weeks	• Matching skills	Baseline, post intervention and after 1 week, 3 weeks, and 5 weeks follow-up	• VMS • Researchers set scales: (Social Validity Measures, Response Accuracy, Task Engagement)	During the intervention, three students maintained a relatively high level of task employment in CAI, compared to TII (Jiahua: PND = 100%; M = 95.3, in CAI; M = 72.5, range 59–82% in TII; Lingling: PND = 83.3%; M = 86.5 in CAI; M = 67.8 in TII; Zicheng: PND = 85.7%; M = 84.1 in CAI; M = 73.4 in TII); all four students required a fewer number of trials to achieve criterion in CAI than in TII; three of the four students required a lower number of prompts and shorter durations in instructional time in CAI than in TII	Statistically significant on some evaluation scales	CAI was more efficient than TII concerning the prompts supplied and the duration of instructional sessions
Macoun SJ et al., 2020, Canada [60]	Journal of Autism and Developmental Disorders	Caribbean Quest; CQ: serious game	PC	Randomized controlled trial	*N* = 20 EG: 11 (9 M, 2 F) CG: 9 (8 M, 1 F) Age: 6–12 yrs	24 sessions, 3 times/week, 30 min per session during 8-weeks	• Attention • Working memory • Academic performance • Inhibitory control	Baseline inter and post intervention	• BRIEF-P T GEC, • Academic supports (tutoring, learning assistance, gifted programming) • Conners-3-P T EF • SRS-2	There was a statistically significant difference in errors (KiTAP ‘Sad/Happy Ghost and Colored Boxes) between the intervention and control groups, F (1,16) = 4.61, *p* < .05, partial η2 = .224; F (1,15) = 12.23, *p* < .01, partial η2 = .49 respectively. On the Woodcock-Johnson Math Fluency task, with posthoc analyses indicating that the intervention group made fewer errors than the control group (Mdiff = 14.08 [95% CI .544–7.61], *p* < .05)	Statistically significant on some evaluation scales	This game has preliminary support and potential efficacy for children with ASD
Mercado J et al.,2018, Mexico [61]	Multimedia Tools and Applications	FarmerKeeper: video game	PC	Randomized controlled trial	*N* = 12 EG: 6 CG: 6 Age: 4–11 yrs	20–30 min, 4-weeks	• Attention • Anxiety	Baseline inter and post intervention	• CRSD-ant test • ADHDT	Participants were 8% (2:00 min approx., *p* = 0.00288) of the time paying more attention during the full session (avg. full session = 13:05 min) when using FarmerKeeper (97.15% of full session) than with BrainCats (89.15% of full session)	Statistically significant at all evaluation scales	FarmerKeeper can improve cognitive indicators by reducing the level of anxiety and increasing the attention of children with autism during the treatment of neurofeedback training sessions
Mercado J et al., 2020, Mexico [30]	Journal on Multimodal User Interfaces	FarmerKeeper: video game	PC	Randomized controlled trial	*N* = 26 EG: 13 CG: 13 Age: 4–13 yrs	13 sessions, 15 min during 10-weeks	• Attention	Baseline inter and post intervention	• CRSD-ant test • ADHDT	There is a slight difference of close to 3% (*p* = 0.25), which means that participants who used FarmerKeeper had a better performance than those who used Cartoons	Statistically significant at all evaluation scales	Pre- and post-assessments revealed that participants' attention, attentional control, and sustained attention were sweetened
Wagle S et al., 2021, India [62]	Scientific Reports	Basket game, Train game, Piano Game, Face game, Shape Game: Serious games	Android playstore	Before & After Clinical Trial (without control)	*N* = 14 EG: 14 (13 M, 1 F) Age: 6–13 yrs	30 min per day during 4 weeks	• Working memory • Facial processing skills	Baseline inter and post intervention	• Corsi-BT • ATEC	No change (W = 7.5, *n* = 14, *P* = 1) was followed in the pre and post-intervention conditions; no significant change was observed in the Corsi total score (W = 27, *n* = 14, *P* = 0.22); participants did not achieve significantly from the month-long game-based training in reducing autistic symptoms or enhancing working memory	Not effective	There is no significant change in the autistic symptoms after the intervention training with the given game
Piana S et al., 2019, Italy [63]	IEEE Transactions on Affective Computing	Guess the Emotion: serious game	PC	Non-randomized Controlled Clinical Trial	*N* = 15 EG: 10 (9 M, 1 F) CG: 5 (4 M, 1 F) Age: 8–11 yrs	10 sessions, 20 min during 4 to 6 weeks	• Emotional Skills • Facial processing skills	Baseline inter and post intervention	• Researchers set scales (Emotions in the recognition task, Response accuracy, Time of response)	There is a significant difference for the recognition, t(9) = 3:984, *p* = 0:003, and the expression task, t(9) = 4:439, *p* = 0:002, indicating that the performance at this task increase significantly from the first to the second period; the MD between the first and the second assessment was lower in the control group (9:60) than in the experimental group (21:50)	Statistically significant at all evaluation scales	During the application of this game, the accuracy of doing the task from the beginning to the end of the training sessions has increased significantly in the trained group compared to the control group
Jouen AL et al., 2017, UK [64]	Child and Adolescent Psychiatry and Mental Health	GOLIAH: Serious Game	PC and Mobile app	Non-randomized Controlled Clinical Trial	*N* = 24 EG: 14 (14 M, 0 F) CG: 10 (10 M, 0 F) Age: 5–8 yrs	100 sessions, 30 min, during 6-months	Attention Imitation skills	Baseline inter and post intervention	• ADI-R • VABS-II • CBCL • SCQ	There were significant signs of progress in ADOS scores, Vineland socialization score, Parental Stress Index total score, and Child Behavior Checklist internalizing, externalizing, and total problems (all *p* < 0.05, Linear Mixed Models, time effect); meaning that treatment given in both groups was favorable and practical	Statistically significant on some evaluation scales	The findings of the 6-month training indicate that both the acceptance of using the game platform and the absence of parental stress have been seen
Hayoung A. Lim et al., 2022, USA [65]	Child Language Teaching and Therapy	An Online Music-Based Speech and Language Learning Game: Online edtech	PC and Mobile app	Before & After Clinical Trial (without control)	*N* = 26 Age: 2–6 yrs	4–6 sessions over a 2-week	Language skills	Baseline and post intervention	• CARS • Researchers set scales (Response accuracy, Time of response, Verbal production)	Outcomes point that the SS4Kids program is an efficacious music-based speech and language training technique for supporting target word production in children across a two-week timespan (all *p* < 0.05.)	Statistically significant at all evaluation scales	Emerging proof of the significance of an online evidence-based practice supports the speech and language outcomes for various children in early intervention
Chaoxin and Jun Yang, 2022, China [66]	Brain sciences	Virtual Training	PC	Randomized controlled trial	*N* = 100, EG1 = 34 EG2 = 33, CG = 33 MAge: 12.9 yrs	3 sess in a week, 30 min	Attention	Baseline, post intervention, and 6-week-follow-up	• CARS • Researchers set scales (Visual attention)	Although none of the three participants noticed progress in the correct rate, the observations of the VT and PE groups were significant (*p* < 0.05) compared to the CG group in uncovering the detection rate of the probe stimulus	Statistically significant at all evaluation scales	This program has the potential to improve visual attention in children with ASD

*Abbreviations*: *SRS-2* Social Responsiveness Scale, *MSEL* Mullen Scales of Early Learning, *WISC-IV* Wechsler Intelligence Scale for Children, *PPVT-R* Peabody Picture Vocabulary Test-Revised, *VABS-II* Vineland Adaptive Behavior Scales-II, *RCPM* Raven’s Colored Progressive Matrices, *GPS* Gaze Positions Scale, *WPPSI* Wechsler Preschool and Primary Scale of Intelligence, *VIQ* Verbal Intelligent Quotient, *PIQ* Performance Intelligent Quotient, *S-CAT* Social-Communication Assessment Tool, *PECS* Picture Exchange Communication System, *ASEBA* Achenbach System of Empirically Based Assessment, *ABC* The Aberrant Behavior Checklist, *SSRS* The Social Skills Rating System, *BRIEF* The Behavior Rating Inventory of Executive Function, *SART* Sustained attention response task, *Corsi-BTT* The Corsi block tapping task, *SSIT* Set-shifting improvement tasks, *WCST* Wisconsin Card Sorting Test, *MCST* Modified Card Sorting Test, *VABS-II* Vineland Adaptive Behavior Scales, *CPM* Colored Progressive Matrices (CPM), Academic Assessments (Math, Reading Comprehension, Copying), *CARS* Childhood Autism Rating Scale, *VMS* Visual Matching Scale, *BRIEF-P T GEC* Behavior Rating Inventory of Executive Function Parent T Score General Executive Composite, *Conners-3-P T EF* Executive Functions, *CRSD-ant test* Attention network test, *ADHDT* Attention-Deficit/Hyperactivity Disorder Test (2nd ed), *ATEC* The Autism Treatment Evaluation Checklist, *ADI-R* Autism Diagnostic Interview-Revised, *CBCL* The Child Behavior Checklist, *SCQ* The Social Communication Questionnaire, *SD* Standard Deviation, *M* Mean, *PC* Personel Computer, *ADOS* Autism Diagnostic Observation Schedule, *CAI* Computer-Assisted Instruction, *TII* Teacher-Implemented Instruction

### Publication analysis

#### Distribution of articles over the past years

**Fig. 3:**
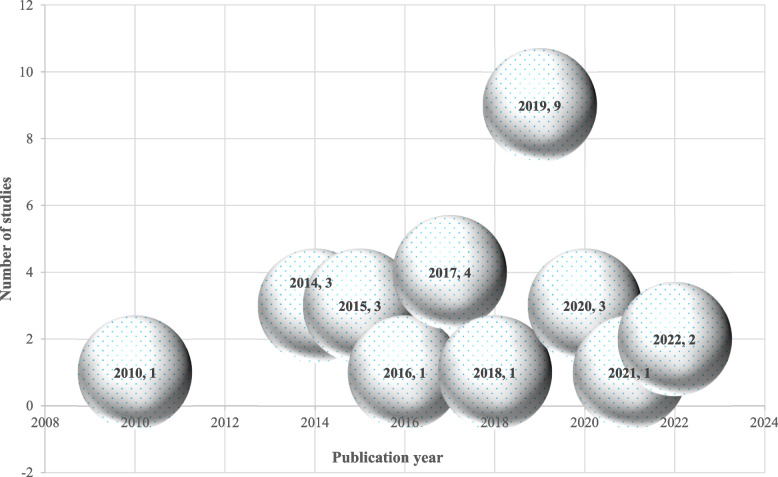
The distribution of included studies based on publication years

The final distribution of citations includes 28 academic papers which met the adjusted inclusion criteria. Figure 3 depicts the frequency of published articles in the time period between 2010 and 2022. As the figure shows, researchers have been interested in applying cognitive games to autism since 2019.

#### Distribution of academic papers by journals, quartile scores, and conference name

Our selected scientific citations (*n* = 28) were retrieved from 25 various journals and one international conference. Most of the reviewed articles have been published in reputable journals. Journal Citation Ranking and Quartile Scores are presented in Table 2. Notably, 17 of 25 journals (68%) are ranked in the first quartile.

**Table 2:** Distribution of papers based on journals, quartile scores, and conference name

**Count of Journal/ Conference name**	**Column Labels**					
**Row Labels**	**Q1**	**Q2**	**Q3**	**Q4**	**Without Q**	**Grand Total**
**Conference**					**1**	**1**
In2015 IEEE Global Engineering Education Conference					1	1
**Journal**	**17**	**5**	**3**	**1**	**1**	**27**
Autism	1					1
Autism Research	1					1
Bio-medical materials and engineering			1			1
Computer Assisted Surgery	1					1
Educational Sciences: Theory & Practice			1			1
European child & adolescent psychiatry	1					1
Frontiers in psychiatry		1				1
IEEE Transactions on Affective Computing	1					1
Indian Journal of Science and Technology				1		1
Information Sciences	1					1
International Journal of Computer Games Technology		1				1
International journal of human–computer interaction	1					1
International Journal of Web-Based Learning and Teaching Technologies		1				1
Journal of Autism and Developmental Disorders	3					3
Journal of Child Psychology and Psychiatry	1					1
Journal of Intellectual Disability Research	1					1
Journal on Multimodal User Interfaces		1				1
Multimedia Tools and Applications	1					1
PRO-FONO: Revista de Actualizacao Cientifica					1	1
Research in Autism Spectrum Disorders		1				1
Scientific Reports	1					1
Virtual Reality	1					1
Child and Adolescent Psychiatry and Mental Health	1					1
Child Language Teaching and Therapy	1					1
Brain sciences			1			1
**Grand Total**	**17**	**5**	**3**	**1**	**2**	**28**

#### Distribution of studies by journal/conference name, publisher and IFs

Based on our results, ProQuest ranked first (19.23%) among the publishers presented in Table 3, while Springer ranked second (11.54%) of the 16 publishers in the current review. It is noteworthy that one of the journals did not have a specific publisher. Table 4 represents the frequency of papers by publishers and journals/conference names and impact factors.

**Table 3:** The frequency of published articles by publishers

Row Labels	Papers	Percentage
Springer, ProQuest	5	19.2%
Springer	3	11.5%
Elsevier	2	7.6%
Sage	2	7.6%
Wiley, ProQuest	2	7.6%
Taylor and Francis	1	7.6%
Frontiers Media S.A	2	3.8%
IGI Publishing	2	3.8%
ProQuest	1	3.8%
Hindawi	1	3.8%
Indian Society for Education and Environment	1	3.8%
John Wiley and Sons Inc	1	3.8%
Nature, ProQuest	1	3.8%
Institute of Electrical and Electronics Engineers Inc	1	3.8%
Edam Egitim Danismanligi	1	3.8%
**Grand Total**	**26**	**100%**

**Table 4:** Distribution of papers based on journal/conference name, publisher and IFs

Journal/Conference name	Journal rank	Impact Factor of journal	Publisher	Count of papers
Autism Research	Q1	5.216	John Wiley and Sons Inc	1
Computer Assisted Surgery	Q1	1.787	Taylor and Francis	1
Educational Sciences: Theory & Practice	Q3	Without IF	Edam Egitim Danismanligi	1
Autism	Q1	5.689	Sage	1
Bio-medical materials and engineering	Q3	1.243	IOS Press	1
International Journal of Computer Games Technology	Q2	Without IF	Hindawi	1
PRO-FONO: Revista de Actualizacao Cientifica	_	Without IF	Unknown	1
In2015 IEEE Global Engineering Education Conference	_	_	_	1
Virtual Reality	Q1	5.095	Springer	1
Frontiers in psychiatry	Q2	4.157	Frontiers Media S.A	1
Indian Journal of Science and Technology	Q4	Without IF	Indian Society for Education and Environment	1
International Journal of Web-Based Learning and Teaching Technologies	Q2	Without IF	IGI Publishing	1
Information Sciences	Q1	6.795	Elsevier	1
Research in Autism Spectrum Disorders	Q2	2.881	Elsevier	1
Journal of Child Psychology and Psychiatry	Q1	8.982	Wiley, ProQuest	1
Journal of Intellectual Disability Research	Q1	2.424	Wiley, ProQuest	1
International journal of human–computer interaction	Q1	3.353	Taylor and Francis	1
European child & adolescent psychiatry	Q1	4.785	Springer, ProQuest	1
Journal of Autism and Developmental Disorders	Q1	4.291	Springer, ProQuest	3
Multimedia Tools and Applications	Q1	2.757	Springer	1
Journal on Multimodal User Interfaces	Q2	1.769	Springer	1
Scientific Reports	Q1	4.379	Nature, ProQuest	1
IEEE Transactions on Affective Computing	Q1	10.506	Institute of Electrical and Electronics Engineers Inc	1
Child and Adolescent Psychiatry and Mental Health	Q1	3.033	Springer, ProQuest	1
Child Language Teaching and Therapy	Q1	1.324	Sage	1
Brain sciences	Q3	3.394	ProQuest	1

#### The distribution of papers by their conducted countries

**Fig. 4:**
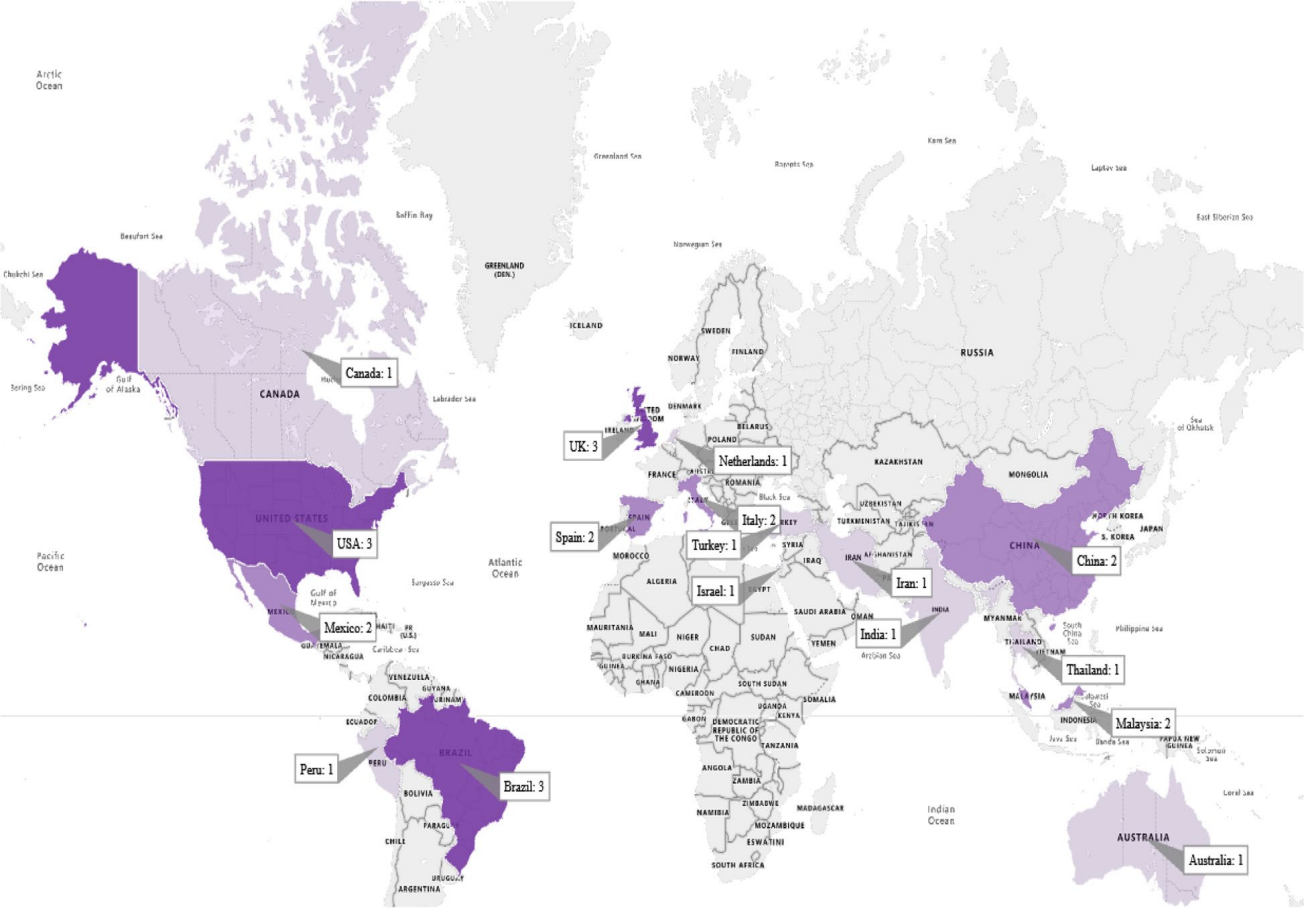
World map displaying the frequency of papers, in which the different color depths outline the different numbers of papers in various countries

The included articles have been published in 17 different countries. The distribution of studies based on country (country of the first author) is shown in Fig. 4 on the worldwide map. As it turns out, USA, UK, and Brazil have the highest frequency compared to other countries.

#### Keywords analysis

**Fig. 5:**
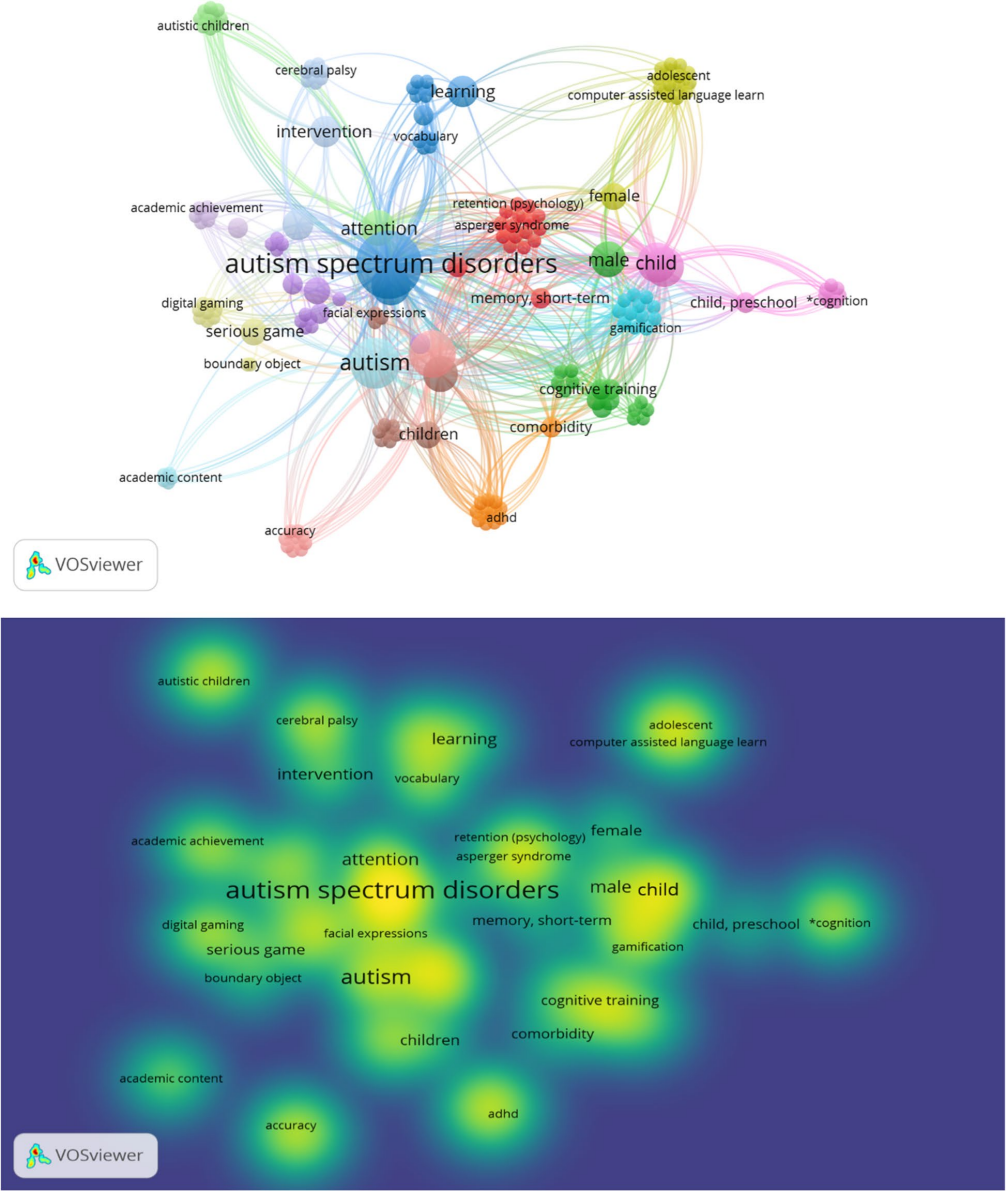
Terms Analysis of included Publications: a) Network Visualization and b) Density Visualization

We performed a co-occurrence analysis based on keywords for included publications. Network visualization and density visualization of terms analyses of the reviewed publications are presented in Fig. 5.

### Methodological quality assessment

**Fig. 6:**
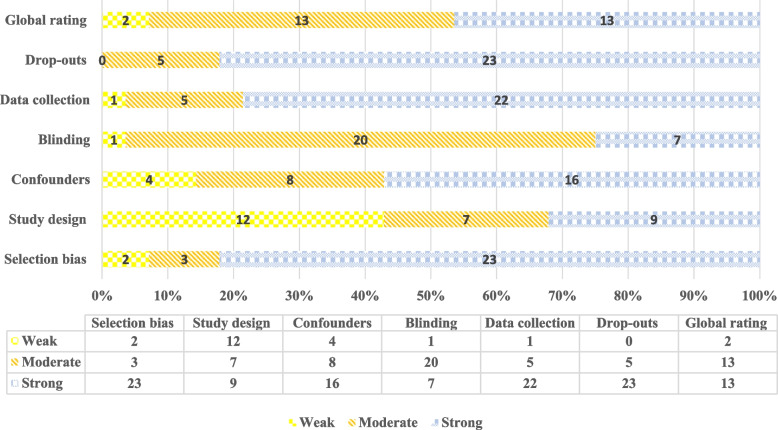
Risk of bias appraisal and quality assessment

Figure 6 provides the methodology quality of the included studies. Based on the sum of scores, most studies were strong in terms of selection bias and drop-outs (82.14%) and moderate in terms of blinding (71.42%). Based on the study design score, 42.85% were weak, and 32.14% were strong. Concerning the global rating score, 46.42% of the reviewed studies were strong, 46.42% moderate, and 7.14% weak.

### Study specifications

#### Frequency of studies by main game types and platforms

In the papers included in our study, serious and video games are the most popular and frequent types of games used in cognitive rehabilitation in autistic children. The deployment platforms for most of the studies were Personal Computers (PC) (*n* = 19, 67.85%) and mobile or tablet applications (android playstore) (*n* = 5, 17.85%).

#### Distribution of papers based on type of studies, sample size and session detail

**Fig. 7:**
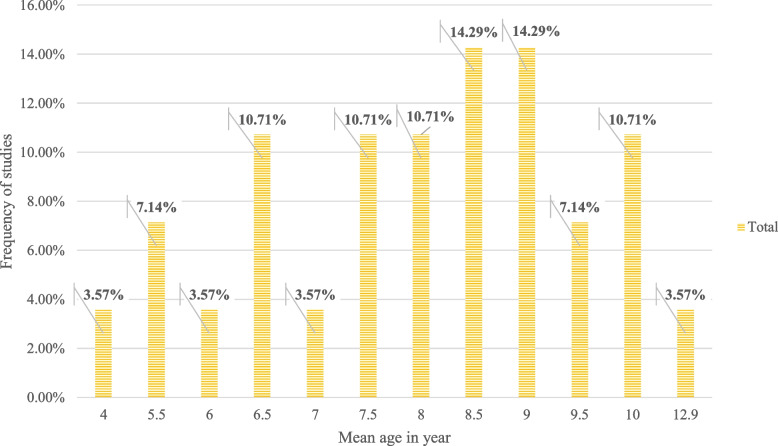
Number of investigations by mean age of participants

In the examined studies, a total of 842 subjects participated; 552 participants were in an Experimental Group (EG), and 290 were in a Control Group (CG). Fifteen studies (53.84%) compared EG vs. CG, while 13 papers (46.42%) evaluated the applied intervention's effect on an experimental group. Out of 28 studies, only 14 papers mentioned the male–female gender ratio; 227 participants (96.59%) were males, and eight participants (3.46%) were females. The youngest and oldest participants were two and 13 years old, respectively. The mean age of the children in the different studies ranged from 4 to 12.9 years ((interquartile range) IQR1: 6. 5, IQR2: 8.25, IQR3: 9). Of the 28 screened studies, the percentage of studies that involved subjects of a specific age range is given in Fig. 7. As can be seen, the age range of 7.5 to 9 years was the most frequent age range for participants. Furthermore, study sample sizes ranged from 4 to 100 participants (IQR1:12, median: 19.5, IQR3: 38). The number of intervention sessions ranged from 1 to 100 sessions, with the time of each session varying (based on minutes); the duration of the interventions varied from one day to 6 months. Interestingly, nine of the screened studies were experimental, and 18 of them were quasi-experimental; the distribution and effectiveness of various study designs are presented in Table 5. It is noteworthy that out of the total number of studies (*n* = 28), 17 studies (60.71%) reported a positive effect on all scales, of which nine studies (52.94%) were quasi-experimental, and seven (41.17%) were true experimental. Totally, only four studies did not report a positive effect.

**Table 5:** Distribution of papers based on study design and effectiveness

Effectiveness: (1. statistically significant, 2. positive without statistical argument, 3.not effective (not statistically significant)) Row Labels	Not effective	Positive without statistical argument	Statistically significant at all evaluation scales	Statistically significant on some evaluation scales	Statistically significant only for some children	Grand Total
**Experimental**			**7**	**2**		**9**
Partially Randomized clinical trial			1			1
Randomized controlled trial			6	2		8
**Quasi-experimental**	**4**	**1**	**9**	**3**	**1**	**18**
Before & After Clinical Trial (without control)	3	1	7	1	1	13
Non-randomized Controlled Clinical Trial	1		2	2		5
**Quasi-experimental& Experimental**			**1**			**1**
Phase 1: Before & After Clinical Trial (without control) Phase 2: Randomized controlled trial with two intervention groups and controls			1			1
**Grand Total**	**4**	**1**	**17**	**5**	**1**	**28**

### Effects of computerized cognitive games on outcomes

Based on the related studies, major domains of cognitive function are divided into six main categories: 1-executive functions, 2- social cognition/emotions, 3- attention/ concentration, 4- learning and memory, 5- language and 6- motor functions [19, 21]. The sixth domain was not included in the current review, because we wanted to focus on the core problems of children with ASD. Hence, we have categorized the targeted cognitive impairments in the reviewed studies (28 studies) in the first five classes. Table 6 presents the effects of cognitive games on autistic children. As can be seen in Table 6, no negative effects of using computer games on the five categories of cognitive outcomes were reported in the included studies.

**Table 6:** Effects of computerized cognitive games on outcomes

Outcome category	Outcomes Subcategory	Effect			
		**Positive**			**No effect**
		^**a**^**SS**	^**b**^**WS**	^**c**^**PS**	
**Executive functions**	Working memory	[22]		[60]	[62]
	Imitation skills	[51–53]		[64]	
	Recognizing and differentiating	[53]			
	Set-shifting ability	[56]			
	Academic performance	[32]		[60]	
	Matching skills			[59]	
	Inhibitory control			[60]	
**Social cognition/emotions**	Social interaction skills	[42, 44]	[50]	[54, 55]	[47]
	Emotional Skills	[50, 58, 63]			[43]
	Facial processing skills	[22, 58, 63]	[50]	[54]	[47, 62]
	Body language	[58]			
	Anxiety	[61]			
**Attention/ concentration**	Attention	[30, 32, 49, 51–53, 61, 66]		[46, 60, 64]	
**Learning and memory**	Learning	[52, 57]			
	Short-term memory	[45, 49]			
	Long-term memory	[45, 49]			
**Language**	Language skills	[65]		[54]	[48]
	Vocal intonation	[58]			

^a^*SS* Statistically significant on all evaluation scales, ^b^WS Positive without statistical argument, and ^c^*PS* Partially Statistically significant (on some evaluation scales or children)

#### Executive functions

Ten studies investigated the effects of cognitive games on executive functions, and nine citations (90%) reported the positive effects of applied games on autistic children in this category. The most positive executive function improvements were related to one subgroup, i.e.; imitation skills (four studies), and in working memory improvement, three studies reported partial effects, and one reported the effects as statistically significant on all evaluation scales.

#### Social cognition/emotions

In 42.85% (12 studies) of the screened papers, social cognition and emotions were examined. The highest rate of effects reported by studies was related to social cognition enhancement. In nine of the studies, the relative and statistical effects of the use of cognitive games on this factor were observed. It should be noted that in some of these studies, some statistical scales were not significant; three studies have reported that functional games were not effective on social cognition.

#### Attention/ concentration

Eleven of the reviewed studies assessed the attention and concentration of children with autism after the use of cognitive games. In most studies (28.57%), positive statistical effects were seen, and some studies reported that effects were relative.

#### Learning, memory and language

Eight of the studies assessed the learning skills, memory, and language skills of children with autism were assessed after the use of cognitive games. Seven studies reported positive effects of applied cognitive games for these categories. The most positive effect of games in these classes was related to the improvement of learning skills and short/long-term memory.

### Reported limitation of studies

Nineteen mined papers (67.85%) reported some limitations and faced fundamental challenges. The limitations mentioned in the studies are provided in Table 7.

**Table 7:** Limitations of included studies

Challenges/limitations	Citations
Sample size was limited	[30, 32, 43, 45, 51, 53, 55, 56, 59–62, 64, 65]
Short-term therapy	[30, 32, 43, 47, 51, 55, 60–62, 65]
Short-term follow-up	[30, 51, 53, 55, 60–62]
Cultural differences and the family environment	[43, 58]
Heterogenous populations	[22, 54]
Lack of control group	[51, 56, 65]
Double-blind design is need	[60]
The lack of more precise and external evaluation of improvements	[51]
Heterogenous populations	[45, 66]
Not blinding assessors	[55]
Double-blind design is need	[32]
Psychotropic medication using	[22]
Study the gender differences	[51]
Limited in developmental profiles, abilities, and difficulties	[45]
Duplication of game objects	[44]
Sensitivity of game pages and children reaction	[44]
Limited generalizability to the wider autism spectrum	[45]
Insert the game in routine program	[47]
Not to randomize experimental treatment	[64]

## Discussion

The main objective of our systematic review was to describe and screen the critical results related to the effectiveness of applying computer-based games for the cognitive rehabilitation of autistic children. In this systematic review, the bibliometric data of the included studies, such as the name of the published journal, its level, and the country of the first author, was analyzed. Moreover,, the specific characteristics of each study, such as the type and the name of the game, platform, sample size, comparison and study group, targeted cognitive functions, evaluation and limitations, are given in detail. This study focused principally on identifying experimental interventions used in digital games to remediate cognitive functions like executive functions, social cognition, attention, face processing, etc. Totally, 28 studies that began to publish and were collected from the WOS, PubMed, Scopus, IEEE Xplore, and APA PsycInfo databases were included for review.

Methodological quality evaluation using the EPHPP scale [41, 67] showed that more than 90% of the studies had a moderate to high-quality design for random controlled experiments, non-random assignment studies, and before and after clinical trials (without control). Of the nine experimental studies, six randomized controlled trials reviewed reported a significant post-intervention effect compared to control groups who did not practice the training game. Two of the randomized controlled studies reported a positive effect on some evaluation scales. Significantly, 17 of the 28 studies reviewed reported positive statistical effects on all evaluation scales; in five studies, positive statistical effects were seen in some evaluation scales, and in one study, a positive effect was seen without performing statistical calculations. In brief, this review recommends that computerized game-based solutions may be helpful in the fields of cognitive rehabilitation and future research in the pediatric population. These issues do not allow any inferences to be made about the potential benefits of game-based interventions beyond more traditional approaches [68, 69].

As 21.42% of the studies included in this review were conducted in the United States and the United Kingdom, it can be concluded that autism in these countries has a relatively higher prevalence compared to the rest of the societies. According to 2020 statistics, about 222 out of every 10,000 children in the United States have ASD, one of the highest prevalence rates in the world [70, 71]. Because of the increased number of children and adolescents with ASD, the United States has tried to develop the most advanced technologies to reduce the problems of these people. The Autism Society of America strives to provide as much perspective as possible for individuals and families with autism [72]. Therefore, studies suggest emerging technologies such as computer games have been designed and widely adopted for people with ASD in developed countries such as the United States [73]. Emerging technologies based on computer games, which have been designed and used in various studies, can significantly affect the cognitive performance of patients and help them achieve relative recovery. Consequently, combining computer technologies and cognitive problems has led investigators in countries such as the United States and the United Kingdom to create computer-based approaches to rehabilitate autistic individuals.

In the screened interventions, various cognitive measures were targeted for rehabilitation using computer-based games. Based on the literature, we classified these cognitive processes, including working memory, attention, social interaction skills, languages, and so on, into five main classes: 1-executive functions, 2- social cognition/emotions, 3- attention/ concentration, 4- learning and memory, and 5- language [21]. Our findings demonstrate that the second metric, i.e., social cognition and emotions, were more commonly considered the main hallmark deficit in autistic children. Clearly, a significant proportion of the studies (48%) targeted the improvement of abilities such as facial processing skills, emotional skills, body language to communicate, etc., as observed in nine studies of relative positive effects in children after the intervention period. Studies have further shown that the use of digital cognitive games can significantly reduce the errors of children with autism in identifying facial expressions (such as sad, happy, surprised, scared, etc.) and improve children's communication to some extent. Similar to our study, a systematic review conducted by Patricia Mesa-Gresa et al. concluded that social cognition problems in children with autism are significant and cognitive games can effectively reduce this impairment [37]. Accordingly, computer-based games offered in a training approach appear to be a hopeful tool for improving the capacity of autistic children to state critical emotions and recognize body motions and gestures [63].

Based on the characteristics of the participants in the studies, the average age range in children was 4 to 12.9 years; however, the age range of 7.5 to 9 years was more considered in the interventions. As autism is more commonly diagnosed in childhood, non-drug therapies can be given more attention at this age. Some of the cognitive problems identified in children with autism require special attention. Therefore, it can be concluded that the use of cognitive rehabilitation approaches at a younger age can be effective in shaping personality and reducing cognitive problems such as executive functions, attention, and working memory deficits [74]. Also, in the mined studied, the participants were mostly boys; as it turns out from the literature, boys are about five times more likely to have autism than girls [75].

The duration of cognitive interventions was varied in our included studies, from ten minutes to an hour in each session. Segers et al. [76] demonstrated that the amount of time children spent at play is related to their improvement in cognitive problems. The results of our review show that the amount of intervention time is also relatively related to the effectiveness of the designed cognitive games. In some of the included studies, as shown in Table 2, the longer the time and greater the number of intervention sessions were, the more positive the effects reported by the researchers were, but this claim is not always accurate. In the few studies we reviewed, although the number of sessions was high, the effectiveness of the game was not observed [43, 47, 48]. The reason for this is related to the target group. Since some autistic children are very vulnerable and doing particular tasks may be disturbing for them, the number of rehabilitation sessions with repetitive games may not be effective for the individual.

Most of the studies in our review (15 out of 28) had a control group to evaluate the effects of cognitive games. It is noteworthy that in some studies, the control group did not receive any intervention, but in others, the control group received other computer games or traditional games instead of the cognitive games of the intervention group [22, 30, 44, 46, 47, 63, 77]. One study had a design that differed from the rest; it had two phases with two intervention groups and one control group [58].

Regarding the type of game and platform, serious games were the most popular type with various avatars and educational scenarios in our included studies. Serious games can also include online options. One of the advantages of serious games is their ability to stimulate behavior and social competition, so playing time increases and, at the same time, motivates children. Serious games can be described as digital/computer games and tools that provide an agenda of educational design and are over entertainment. However, some games included in this review can be executed online. Online games can allow the therapist to monitor the patients remotely, control their progress, and adjust the game’s goals. In line with our review, one study [78] noted that the use of serious online games could lead to easier control of children, and the therapist could remotely monitor the child's progress and performance. Notably, most studies used the PC platform for games. This platform includes Mac, Sony, Asus, HP, etc. Because most cognitive rehabilitation studies were performed in medical centers, laptops or computers were used.

Computerized game-based training solutions have advantages for cognition remedies beyond traditional therapies. These rehabilitation approaches have the capacity to simulate various imaginary or real-life conditions. Furthermore, unlike conventional training methods, they present a peaceful and safe setting for limitless repetition of training duties and better performance [79].

Based on the results of this systematic study, the included studies have major limitations and challenges that cannot be ignored. One of the most significant challenges was the limited sample size for evaluating the effectiveness of cognitive games. Because communicating with autistic children is difficult and challenging, and these children have an unknown spectrum of behavior [56], their parents resist entering intervention studies or refuse to cooperate after entering the study. Short-term treatment or intervention and short-term follow-up are other reported limitations. The cost of intervention studies and the refusal of further cooperation by children's parents are among the reasons for these limitations.

### Limitations and strengths of this study

The current study has both strengths and weaknesses. The strengths of the study comprise:

- Application of an extensive search strategy to identify a large number of studies (1746 studies),
- Performance of searches to retrieve studies in five important databases, namely WOS, Scopus, Medline (through PubMed), IEEE Xplore, and APA PsycInfo,
- Review and evaluation of studies to extract data by three authors independently,
- Use of comprehensive tools for evaluating the quality of included studies,
- Performance of a manual search to retrieve possible missing studies.

Some limitations were also encountered in this study, particularly the challenges to comparing studies caused by the heterogeneity of the results and the exclusion of studies published in languages other than English.

## Conclusion

This qualitative review spotlights the use of various computerized games to enhance cognitive metrics in autistic children. Employing a systematic approach, the authors have provided a comprehensive overview of the usage of cognitive games that could rehabilitate factors like executive function, attention, memory, daily skills, and social cognition. This review study demonstrated that computer games have the potential and effectiveness to improve the cognition of children with ASD. At the same time, the findings of this review could encourage investigators to utilize new comprehensive methods to remedy the defects of people with ASD, especially at a young age. Nevertheless, more studies are required to examine the real effects of these technologies and their effectiveness.

## Supplementary Information

**Additional file 1: Table A.1.** Keywords and search strategy for each database.

## Data Availability

All data generated or analyzed during this study are included in this published article.

## References

[1] Faras H, Al Ateeqi N, Tidmarsh L (2010). Autism spectrum disorders. Ann Saudi Med.

[2] Licari MK, Alvares GA, Varcin K, Evans KL, Cleary D, Reid SL (2020). Prevalence of motor difficulties in autism spectrum disorder: analysis of a population-based cohort. Autism Res.

[3] Maenner MJ, Shaw KA, Baio J, Washington A, Patrick M, DiRienzo M, Christensen DL, Wiggins LD, Pettygrove S, Andrews JG, Lopez M (2020). Prevalence of autism spectrum disorder among children aged 8 years—autism and developmental disabilities monitoring network, 11 sites, United States, 2016. MMWR Surveill Summ.

[4] Bilder DA, Bakian AV, Stevenson DA, Carbone PS, Cunniff C, Goodman AB, McMahon WM, Fisher NP, Viskochil D (2016). Brief report: the prevalence of neurofibromatosis type 1 among children with autism spectrum disorder identified by the autism and developmental disabilities monitoring network. J Autism Dev Disord.

[5] Bal VH, Wilkinson E, Fok M (2022). Cognitive profiles of children with autism spectrum disorder with parent-reported extraordinary talents and personal strengths. Autism.

[6] Rommelse N, Langerak I, Van Der Meer J, De Bruijn Y, Staal W, Oerlemans A (2015). Intelligence may moderate the cognitive profile of patients with ASD. PLoS ONE.

[7] Joseph RM, Tager-Flusberg H, Lord C (2002). Cognitive profiles and social-communicative functioning in children with autism spectrum disorder. J Child Psychol Psychiatry.

[8] Taylor LJ, Maybery MT, Grayndler L, Whitehouse AJ (2014). Evidence for distinct cognitive profiles in autism spectrum disorders and specific language impairment. J Autism Dev Disord.

[9] Long C, Gurka MJ, Blackman J (2011). Cognitive skills of young children with and without autism spectrum disorder using the BSID-III. Autism Res Treat.

[10] Dawson G, Munson J, Estes A, Osterling J, McPartland J, Toth K (2002). Neurocognitive function and joint attention ability in young children with autism spectrum disorder versus developmental delay. Child Dev.

[11] Kuschner ES, Bennetto L, Yost K (2007). Patterns of nonverbal cognitive functioning in young children with autism spectrum disorders. J Autism Dev Disord.

[12] Dhamodharan T, Thomas M, Ramdoss S, JothiKumar K, SaravanaSundharam S, Muthuramalingam B, Hussainalikhan N, Ravichandran S, Vadivel V, Suresh P, Buddhan S. Cognitive rehabilitation for autism children mental status observation using virtual reality based interactive environment. InInternational Conference on Intelligent Human Systems Integration 2020 Feb 19 (pp. 1213–1218). Springer, Cham.

[13] Helt M, Kelley E, Kinsbourne M, Pandey J, Boorstein H, Herbert M (2008). Can children with autism recover? if so, how?. Neuropsychol Rev.

[14] Eack SM, Hogarty SS, Greenwald DP, Litschge MY, Porton SA, Mazefsky CA (2018). Cognitive enhancement therapy for adult autism spectrum disorder: results of an 18-month randomized clinical trial. Autism Res.

[15] Mandy W, Murin M, Skuse D. The cognitive profile in autism spectrum disorders. Autism spectrum disorders. 180: Karger Publishers; 2015. p. 34–45.

[16] Rommelse NN, Geurts HM, Franke B, Buitelaar JK, Hartman CA (2011). A review on cognitive and brain endophenotypes that may be common in autism spectrum disorder and attention-deficit/hyperactivity disorder and facilitate the search for pleiotropic genes. Neurosci Biobehav Rev.

[17] Pellicano E, Maybery M, Durkin K, Maley A (2006). Multiple cognitive capabilities/deficits in children with an autism spectrum disorder:“Weak” central coherence and its relationship to theory of mind and executive control. Dev Psychopathol.

[18] Englund JA, Decker SL, Allen RA, Roberts AM (2014). Common cognitive deficits in children with attention-deficit/hyperactivity disorder and autism: working memory and visual-motor integration. J Psychoeduc Assess.

[19] Palta P, Schneider AL, Biessels GJ, Touradji P, Hill-Briggs F (2014). Magnitude of cognitive dysfunction in adults with type 2 diabetes: a meta-analysis of six cognitive domains and the most frequently reported neuropsychological tests within domains. J Int Neuropsychol Soc.

[20] Jin P, Wang Y, Li Y, Xiao Y, Li C, Qiu N, Weng J, Fang H, Ke X (2020). The fair decision-making of children and adolescents with high-functioning autism spectrum disorder from the perspective of dual-process theories. BMC Psychiatry.

[21] Sachdev PS, Blacker D, Blazer DG, Ganguli M, Jeste DV, Paulsen JS (2014). Classifying neurocognitive disorders: the DSM-5 approach. Nat Rev Neurol.

[22] de Vries M, Prins PJ, Schmand BA, Geurts HM (2015). Working memory and cognitive flexibility-training for children with an autism spectrum disorder: a randomized controlled trial. J Child Psychol Psychiatry.

[23] Cicerone KD, Dahlberg C, Malec JF, Langenbahn DM, Felicetti T, Kneipp S (2005). Evidence-based cognitive rehabilitation: updated review of the literature from 1998 through 2002. Arch Phys Med Rehabil.

[24] Plichta P (2018). The use of information and communication technologies by young people with intellectual disabilities in the context of digital inequalities and digital exclusion. E-methodology.

[25] Desideri L, Di Santantonio A, Varrucciu N, Bonsi I, Di Sarro R (2020). Assistive technology for cognition to support executive functions in autism: a scoping review. Adv Neurodev Disord.

[26] Mohd CK, Shahbodin F, Sedek M, Samsudin M (2020). Game based learning for autism in learning mathematics. Int J Adv Sci Technol.

[27] Rahman MR, Naha S, Roy PC, Ahmed I, Samrose S, Rahman MM, Ahmed SI. A-class: A classroom software with the support for diversity in aptitudes of autistic children. In2011 IEEE Symposium on Computers & Informatics 2011 Mar 20 (pp. 727–731). IEEE.

[28] Vallefuoco E, Bravaccio C, Pepino A. Serious games in autism spectrum disorder-an example of personalised design. InSpecial Session on Serious Games on Computer Science Learning 2017 Apr 21 (Vol. 2, pp. 567–572). SciTePress.

[29] Blumberg FC, Deater-Deckard K, Calvert SL, Flynn RM, Green CS, Arnold D, Brooks PJ. Digital games as a context for children’s cognitive development: Research recommendations and policy considerations. Soc Policy Rep. 2019;32(1):1–33.

[30] Mercado J, Escobedo L, Tentori M (2021). A BCI video game using neurofeedback improves the attention of children with autism. J. Multimodal User Interfaces.

[31] Shahmoradi L, Mohammadian F, Rahmani Katigari M (2022). A systematic review on serious games in attention rehabilitation and their effects. Behav Neurol.

[32] Spaniol MM, Shalev L, Kossyvaki L, Mevorach C (2018). Attention training in autism as a potential approach to improving academic performance: a school-based pilot study. J Autism Dev Disord.

[33] Shahmoradi L, Rezayi S (2022). Cognitive rehabilitation in people with autism spectrum disorder: a systematic review of emerging virtual reality-based approaches. J Neuroeng Rehabil.

[34] Dechsling A, Orm S, Kalandadze T, Sütterlin S, Øien RA, Shic F, et al. Virtual and augmented reality in social skills interventions for individuals with autism spectrum disorder: A scoping review. Journal of autism and developmental disorders. 2021:1–16. 10.1007/s10803-021-05338-5 PMC9556391 34783991

[35] Liu X, Wu Q, Zhao W, Luo X (2017). Technology-facilitated diagnosis and treatment of individuals with autism spectrum disorder: an engineering perspective. Appl Sci.

[36] van Bennekom MJ, de Koning PP, Denys D (2017). Virtual reality objectifies the diagnosis of psychiatric disorders: a literature review. Front Psychiatry.

[37] Mesa-Gresa P, Gil-Gómez H, Lozano-Quilis JA, Gil-Gómez JA (2018). Effectiveness of virtual reality for children and adolescents with autism spectrum disorder: an evidence-based systematic review. Sensors.

[38] Zhang X, Tan R, Lam WC, Yao L, Wang X, Cheng CW, Liu F, Chan JC, Aixinjueluo Q, Lau CT, Chen Y (2020). PRISMA (preferred reporting items for systematic reviews and meta-analyses) extension for Chinese herbal medicines 2020 (PRISMA-CHM 2020). Am J Chin Med.

[39] Mesa-Gresa P, Gil-Gómez H, Lozano-Quilis J-A, Gil-Gómez J-A (2018). Effectiveness of virtual reality for children and adolescents with autism spectrum disorder: an evidence-based systematic review. Sensors.

[40] Cochrane. Non-randomised Controlled Study (NRS) Designs. Cochrane Childhood Cancer Amsterdam; 2019.

[41] Thomas B, Ciliska D, Dobbins M, Micucci S. Quality assessment tool for quantitative studies dictionary: the Effective Public Health Practice Project (EPHPP). McMaster University. 2008.

[42] Alvares GA, Chen NT, Notebaert L, Granich J, Mitchell C, Whitehouse AJ (2019). Brief social attention bias modification for children with autism spectrum disorder. Autism Res.

[43] Chen J, Wang G, Zhang K, Wang G, Liu L (2019). A pilot study on evaluating children with autism spectrum disorder using computer games. Comput Hum Behav.

[44] Özen A. Effectiveness of siblings-delivered iPad game activities in teaching social interaction skills to children with autism spectrum disorders. Educational Sciences: Theory & Practice. 2015;15(5).

[45] Fantasia V, Markant DB, Valeri G, Perri N, Ruggeri A (2020). Memory enhancements from active control of learning in children with autism spectrum disorder. Autism.

[46] Aresti-Bartolome N, Garcia-Zapirain B (2015). Cognitive rehabilitation system for children with autism spectrum disorder using serious games: a pilot study. Bio-Med Mater Eng.

[47] Almeida LM, Silva DP, Theodório DP, Silva WW, Rodrigues SC, Scardovelli TA, Silva AP, Bissaco MA (2019). ALTRIRAS: a computer game for training children with autism spectrum disorder in the recognition of basic emotions. Int J Computer Games Technol.

[48] Fernandes FD, Santos TH, Amato CA, Molini-Avejonas DR (2010). Computerized resources in language therapy with children of the autistic spectrum. Pro-Fono.

[49] Al-Hammadi M, Abdelazim A, editors. Randomness impact in digital game-based learning. 2015 IEEE Global Engineering Education Conference (EDUCON); 2015 18–20 March 2015.

[50] Pedreschi VB, Díaz DA, Aguirre JA, Gonzalez PA. A technological platform using serious game for children with Autism Spectrum Disorder (ASD) in Peru. Virtual Reality.;16:17.

[51] Bono V, Narzisi A, Jouen AL, Tilmont E, Hommel S, Jamal W, Xavier J, Billeci L, Maharatna K, Wald M, Chetouani M (2016). GOLIAH: a gaming platform for home-based intervention in autism–principles and design. Front Psych.

[52] Kamaruzaman NN, Jomhari N, Kamarulzaman N, Yusoff M (2016). Engaging children with severe autism in learning Al-Quran through the serious game. Indian J Sci Technol.

[53] Jeekratok K, Chanchalor S, Murphy E (2014). Web-based social stories and games for children with autism. Int J Web-Based Learning and Teaching Technologies.

[54] Bernardini S, Porayska-Pomsta K, Smith TJ (2014). ECHOES: An intelligent serious game for fostering social communication in children with autism. Inf Sci.

[55] Mairena MÁ, Mora-Guiard J, Malinverni L, Padillo V, Valero L, Hervás A, Pares N (2019). A full-body interactive videogame used as a tool to foster social initiation conducts in children with Autism Spectrum Disorders. Res Autism Spectrum Disord.

[56] Saniee S, Pouretemad HR, Zardkhaneh SA (2019). Developing set-shifting improvement tasks (SSIT) for children with high-functioning autism. J Intellect Disabil Res.

[57] Khowaja K, Salim SS (2020). A framework to design vocabulary-based serious games for children with autism spectrum disorder (ASD). Univ Access Inf Soc.

[58] Fridenson-Hayo S, Berggren S, Lassalle A, Tal S, Pigat D, Meir-Goren N, O’Reilly H, Ben-Zur S, Bölte S, Baron-Cohen S, Golan O (2017). ‘Emotiplay’: a serious game for learning about emotions in children with autism: results of a cross-cultural evaluation. Eur Child Adolesc Psychiatry.

[59] Hu XY, Lee GT, Tsai YT, Yang Y, Cai S (2020). Comparing Computer-Assisted and Teacher-Implemented Visual Matching Instruction for Children with ASD and/or Other DD. J Autism Dev Disord.

[60] Macoun SJ, Schneider I, Bedir B, Sheehan J, Sung A (2021). Pilot study of an attention and executive function cognitive intervention in children with autism spectrum disorders. J Autism Dev Disord.

[61] Mercado J, Espinosa-Curiel I, Escobedo L, Tentori M (2019). Developing and evaluating a BCI video game for neurofeedback training: the case of autism. Multimedia Tools and Applications.

[62] Wagle S, Ghosh A, Karthic P, Ghosh A, Pervaiz T, Kapoor R, Patil K, Gupta N (2021). Development and testing of a game-based digital intervention for working memory training in autism spectrum disorder. Sci Rep.

[63] Piana S, Malagoli C, Usai MC, Camurri A (2019). Effects of computerized emotional training on children with high functioning autism. IEEE Trans Affect Comput.

[64] Jouen AL, Narzisi A, Xavier J, Tilmont E, Bodeau N, Bono V, Ketem-Premel N, Anzalone S, Maharatna K, Chetouani M, Muratori F (2017). GOLIAH (Gaming Open Library for Intervention in Autism at Home): a 6-month single blind matched controlled exploratory study. Child Adolesc Psychiatry Ment Health.

[65] Lim HA, Ellis EM, Sonnenschein D. Effect of Sing and Speak 4 Kids: An Online Music-Based Speech and Language Learning Game for Children in Early Intervention. Child Language Teaching and Therapy. 2022:02656590221080308.

[66] Ji C, Yang J (2021). Effects of physical exercise and virtual training on visual attention levels in children with autism spectrum disorders. Brain Sci.

[67] Armijo-Olivo S, Stiles CR, Hagen NA, Biondo PD, Cummings GG (2012). Assessment of study quality for systematic reviews: a comparison of the cochrane collaboration risk of bias tool and the effective public health practice project quality assessment tool: methodological research. J Eval Clin Pract.

[68] Alashram AR, Annino G, Padua E, Romagnoli C, Mercuri NB (2019). Cognitive rehabilitation post traumatic brain injury: a systematic review for emerging use of virtual reality technology. J Clin Neurosci.

[69] Neugnot-Cerioli M, Gagner C, Beauchamp MH (2015). The use of games in paediatric cognitive intervention: A systematic review. International Journal of Physical Medicine & Rehabilitation.

[70] Coury DL, Murray DS, Fedele A, Hess T, Kelly A, Kuhlthau KA (2020). The autism treatment network: bringing best practices to all children with autism. Pediatrics..

[71] Kiseleva M, Yagovkina L, Ovsyannikova A, Baranov S. Statistical Analysis of the Prevalence of Persons with Autism in Modern Society. InEcological-Socio-Economic Systems: Models of Competition and Cooperation (ESES 2019) 2020;16 (pp. 582–586). Atlantis Press.

[72] Hurlbutt K, Chalmers L (2002). Adults with autism speak out: perceptions of their life experiences. Focus Autism Other Dev Disabl.

[73] Valencia K, Rusu C, Quiñones D, Jamet E (2019). The impact of technology on people with autism spectrum disorder: a systematic literature review. Sensors.

[74] Weitlauf AS, Broderick N, Stainbrook JA, Taylor JL, Herrington CG, Nicholson AG, Santulli M, Dykens EM, Juárez AP, Warren ZE (2020). Mindfulness-based stress reduction for parents implementing early intervention for autism: An RCT. Pediatrics..

[75] Jo H, Eckel SP, Wang X, Chen J-C, Cockburn M, Martinez MP (2019). Sex-specific associations of autism spectrum disorder with residential air pollution exposure in a large Southern California pregnancy cohort. Environ Pollut.

[76] Segers E, Verhoeven L (2005). Long-term effects of computer training of phonological awareness in kindergarten. J Comput Assist Learn.

[77] Yerys BE, Bertollo JR, Kenworthy L, Dawson G, Marco EJ, Schultz RT (2019). Brief report: Pilot study of a novel interactive digital treatment to improve cognitive control in children with autism spectrum disorder and co-occurring ADHD symptoms. J Autism Dev Disord.

[78] Zayeni D, Raynaud J-P, Revet A (2020). Therapeutic and Preventive Use of Video Games in Child and Adolescent Psychiatry: A Systematic Review. Front Psych.

[79] Stewart J, Bleumers L, Van Looy J, Mari쮠I, All A, Schurmans D, Willaert K, De Grove F, Jacobs A, Misuraca G, authors Centeno Mediavilla I, editor. The Potential of Digital Games for Empowerment and Social Inclusion of Groups at Risk of Social and Economic Exclusion: Evidence and Opportunity for Policy\r\n. EUR 25900. Luxembourg (Luxembourg): Publications Office of the European Union; 2013. JRC78777.

