# Supplementary material for: Features and effects of computer-based games on cognitive impairments in children with autism spectrum disorder: an evidence-based systematic literature review
Source: BMC Psychiatry. 2023 Jan 3;23:2. doi: 10.1186/s12888-022-04501-1 (PMC9809031; doi:10.1186/s12888-022-04501-1)
Supplement: Supplementary file 1 — Additional file 1: Table A.1. Keywords and search strategy for each database. [file 12888_2022_4501_MOESM1_ESM.docx]

**Appendix A**

**Table A.1.** Keywords and search strategy for each database

| **Database** | **Search strategy** |
| --- | --- |
| **PubMed** | ("Video Games"[Mesh] OR "Game" OR "Games" OR "Gamification" OR "Video Game" OR "Computer Games" OR "Computer Game") AND ( "Autistic Disorder"[Mesh] OR "Autism Spectrum Disorder"[Mesh] OR “Autism” OR“ Autistic child” OR “Autistic children” OR “Autistic disorder” OR “ Autistic spectrum disorder” OR “Classical autism” OR “Early infantile autism” OR “Infantile autism” OR "Typical autism”) AND ("Cognition" OR "Cognition Therapy" OR "Cognitive Dysfunction" OR "Cognitive Decline" OR "Cognitive Impairment" OR "cognitive task" OR "cognitive thinking" OR "cognitive rehabilitation" OR "Cognitive function" OR “attention” OR “Executive function” OR “Confusion” OR “imagination” OR “ learning” OR “ memory” OR “orientation” OR “thinking” OR “numerical cognition” OR “cognitive reserve” OR “social cognition”) AND ("Child"[Mesh] OR "Children" OR "Child, Preschool"[Mesh] OR "Preschool Child" OR "Children, Preschool" OR "Preschool Children" )  Results=112 |
| **Web of Science** | TS=("Video Games" OR "Game" OR "Games" OR "Gamification" OR "Video Game" OR "Computer Games" OR "Computer Game") AND TS=( "Autistic Disorder" OR "Autism Spectrum Disorder" OR “Autism” OR “ Autistic child” OR “Autistic children” OR “Autistic disorder” OR “ Autistic spectrum disorder” OR “Classical autism” OR “Early infantile autism” OR “Infantile autism” OR "Typical autism") AND TS=("Cognition" OR "Cognition Therapy" OR "Cognitive Dysfunction" OR "Cognitive Decline" OR "Cognitive Impairment" OR "cognitive task" OR "cognitive thinking" OR "cognitive rehabilitation" OR "Cognitive function" OR “attention” OR “executive function” OR “confusion” OR “imagination” OR “learning” OR “memory” OR “orientation” OR “thinking” OR “numerical cognition” OR “cognitive reserve” OR “social cognition” ) AND TS=("Child" OR "Children " OR "Preschool Child" OR "Preschool Children")  Results= 441 |
| **Scopus** | TITLE-ABS-KEY-AUTH ( ( "Video Games " OR "Game" OR "Games" OR "Gamification" OR "Video Game" OR "Computer Games" OR "Computer Game" ) AND ( "Autistic Disorder" OR "Autism Spectrum Disorder" OR "Autism" OR " Autistic child" OR "Autistic children" OR "Autistic disorder" OR " Autistic spectrum disorder" OR "Classical autism" OR "Early infantile autism" OR "Infantile autism" OR "Typical autism" ) AND ( "Cognition" OR "Cognition Therapy" OR "Cognitive Dysfunction" OR "Cognitive Decline" OR "Cognitive Impairment" OR "cognitive task" OR "cognitive thinking" OR "cognitive rehabilitation" OR "Cognitive function" OR "attention" OR "executive function" OR "confusion" OR "imagination" OR "learning" OR "memory" OR "orientation" OR "thinking" OR "numerical cognition" OR "cognitive reserve" OR “Social cognition”) AND ( "Child" OR "Children" OR "Preschool Child" OR "Preschool Children" ) )  Results= 568 |
| APA PsycInfo | ("Video Games “OR "Game" OR "Games" OR "Gamification" OR "Video Game" OR "Computer Games" OR "Computer Game") AND ( "Autistic Disorder" OR "Autism Spectrum Disorder" OR “Autism” OR “ Autistic child” OR “Autistic children” OR “Autistic disorder” OR “ Autistic spectrum disorder” OR “Classical autism” OR “Early infantile autism” OR “Infantile autism” OR "Typical autism") AND ("Cognition" OR "Cognition Therapy" OR "Cognitive Dysfunction" OR "Cognitive Decline" OR "Cognitive Impairment" OR "cognitive task" OR "cognitive thinking" OR "cognitive rehabilitation" OR "Cognitive function" OR “attention” OR “executive function” OR “confusion” OR “imagination” OR “learning” OR “memory” OR “orientation” OR “thinking” OR “numerical cognition” OR “cognitive reserve” OR “social cognition” ) AND ("Child" OR "Children” OR "Preschool Child" OR "Preschool Children" ) Excluded: Dissertations, Books= 358  Results= 358 |
| IEEE Xplore | ("All Metadata":Gam*) AND ("All Metadata":Cognit* OR "All Metadata":attention OR "All Metadata":"Executive function" OR "All Metadata":Confusion OR "All Metadata":imagination OR "All Metadata":learning OR "All Metadata":memory OR "All Metadata":orientation OR "All Metadata":thinking) AND ("All Metadata":Autis*) AND ("All Metadata":Child*)  Results=154 |
